# SPP1+ tumor-associated macrophages define a high-risk subgroup and inform personalized therapy in hepatocellular carcinoma

**DOI:** 10.3389/fonc.2025.1606195

**Published:** 2025-07-01

**Authors:** Wei-Xuan Xu, Ya-Mei Ye, Jia-Lin Chen, Xin-Ying Guo, Chen Li, Juan Luo, Lin-Bin Lu, Xiong Chen

**Affiliations:** ^1^ Department of Oncology, Mengchao Hepatobiliary Hospital of Fujian Medical University, Fuzhou, Fujian, China; ^2^ Department of Hepatology, Mengchao Hepatobiliary Hospital of Fujian Medical University, Fuzhou, Fujian, China

**Keywords:** hepatocellular carcinoma, tumor-associated macrophages, single-cell RNA-seq, patient stratification, clinical treatment

## Abstract

**Introduction:**

Recently, contrary to attacking cancer cells, the tumor microenvironment (TME) with genomic stability and vulnerable nature has emerged as promising therapeutic targets in hepatocellular carcinoma (HCC). Within TME ecosystem, tumor-associated macrophages (TAMs) play a pivotal role in tumor evasion and progression of HCC. However, their clinical and therapeutic implications remain unexplored.

**Methods:**

Utilizing a large-scale sc-RNA seq dataset, a landscape of HCC cellular ecosystem was depicted. Based on previous literature, an effectively differential TAMs subset classification was identified. Gene variations was extracted through trajectory analysis and then unsupervised clustering was conducted within RNA-seq data. Subsequently, survival analysis, specific pathway enrichment as well as hub regulatory network analysis were performed. Additionally, the immune cell infiltration and genomic variations were evaluated between clusters. Drug sensitivity and underlying therapeutic molecular were also explored. Through multiple immunofluorescence, our findings were verified.

**Results:**

Herein, integrating single-cell RNA sequencing (scRNA-seq) and bulk RNA-seq data, we established a novel TAM classification system based on mutually exclusive *SPP1* and *FOLR2* signatures. According to the TAM trajectory genes, unsupervised clustering stratified HCC into three distinct clusters. Cluster 3 (C3), which is characterized by metabolic dysregulation and immunosuppressive TME, exhibited the poorest prognosis among the three groups. Hub network analysis of C3 further indicated its characteristic dysregulation of liver-specific metabolism. *SPP1* was identified as a key signature of C3, which contributed to suppressing the infiltration of CD8^+^ T cells. Therapeutic evaluation revealed that C3 were sensitive to chemotherapy and tyrosine kinase inhibitors, while those C1 and C2 were more suitable for immunotherapy. Drug screening identified potential therapeutic compounds for each cluster.

**Conclusion:**

This study redefines the heterogeneity of TAMs beyond the M1/M2 paradigm, linking the TAMs trajectory genes to HCC patient stratification. *SPP1* blockade emerged as a strategy for counteracting immunosuppression, and cluster-specific therapies may optimize the management of HCC.

## 1 Introduction

Liver cancer has the sixth highest incidence and is ranked as the third leading cause of malignant mortality worldwide (1, 2), with hepatocellular carcinoma (HCC) accounting for 85%–90% (3, 4). According to the patients’ tumor stage, liver function, and performance status, a wide range of treatment options, including organ transplantation, surgical resection, radiation, and transarterial and systemic therapies, are alternatives for HCC (4, 5). With the approval of new agents as first- and second-line therapies, in particular the combination of atezolizumab with bevacizumab (T+A) as frontline standard care, the survival outcomes are superior than ever before (6, 7). Nevertheless, the prognosis remains dismal. Owing to the discouraging objective response rate, therapy resistance, and the high relapse probability, the overall 5-year survival rate is limited to 18% (8–10). Moreover, HCC displays high complexity and heterogeneity. Patients in the same clinical stage can respond differently to the same therapy (5, 11). Although unprecedented efforts based on multi-omics have been made for the molecular stratification of HCC, there is still a lack of a simple classification deriving from a single variable or molecular biomarker for effective clinical practice.

Currently, contrary to directly attacking cancer cells, the tumor microenvironment (TME) with genomic stability and vulnerable nature has increasingly emerged as a promising therapeutic target (12, 13). Among the cellular ecosystems of solid malignancy, tumor-associated macrophages (TAMs) play a pivotal role in governing cellular and molecular interactions, sustaining hallmarks of cancer, shaping TMEs, and eventually mapping onto clinical outcomes, as elucidated in studies (14, 15). Although the conventional M1/M2 macrophage classification simply and effectively defined the function and differentiation state of macrophages *in vitro* (13, 16), accumulating evidence based on single-cell RNA sequencing (scRNA-Seq) has challenged the applicability of this dichotomy for complex macrophages *in vivo*, particularly the TAMs (17, 18). Although multiple high resolutions of TAM subsets have been defined by scRNA-seq, the question of how to classify TAMs based on a relevant yet simple variable remains unanswered. It is well known that *SPP1* encodes osteopontin (OPN), a phosphorylated glycoprotein regarded as a key component for tumor cell evolution and microenvironment reprogramming (19). Recently, SPP1+ macrophages have been recognized in several types of tumors and tend to present the malignant polarity of TAMs. The intratumoral cellular programs of SPP1+ TAMs, including promoting angiogenesis, enhancing tumor cell invasion, and resisting immune checkpoint blockade (ICB) therapy, have been reported (13, 14, 20–22). Nevertheless, how to define the clinical phenotypes and guide population-oriented therapy for HCC using SPP1+ TAM signatures has received little attention. There is an urgent need for the simple stratification of patients with HCC and the promotion of subset-targeted interventions.

scRNA-seq is a powerful technology for characterizing the heterogeneity of complex biological systems (23, 24). As a counterpart, the traditional RNA-seq approach, which considers the tumor but not the cell as a unit, obtains the average gene expression and individual phenotype (25). Trajectory analysis is widely used for cell differentiation inference and transcriptome dynamic process decoding (26). In particular, compared with superficial differential expression gene analysis that is conducted at a cluster resolution, trajectory analysis provides an effective way to explore intricate cell-to-cell variations at a single-cell resolution (13, 27). Herein, we depict a landscape of the cellular ecosystem of HCC utilizing a large-scale scRNA-seq dataset and identify a novel differential TAM subset classification for SPP1+ and FOLR2+ macrophages. Through trajectory analysis, we extract the underlying gene variations and define the TAM-based molecular classification of HCC, termed subgroups C1, C2, and C3, within the RNA-seq data. Furthermore, we explore the survival analysis, biological characteristics, hub regulatory gene network, and the genome variations between these three subgroups. We also evaluate the drug sensitivity and identify the underlying therapeutic molecules for each cluster. Accordingly, subtype-specific therapeutic strategies are proposed.

## 2 Materials and methods

### 2.1 Data acquisition and processing

A total of six independent cohorts were collected and processed in this study. The HCC scRNA-seq dataset GSE149614 was downloaded from the Gene Expression Omnibus (GEO). Excluding eight normal liver tissues (NLTs), we retained 10 primary tumor (PT), two portal vein tumor thrombus (PVTT), and one metastatic lymph node (MLN) samples (28). The Cancer Genome Atlas liver cancer cohort (TCGA-LIHC) and corresponding phenotype information, including 335 tumor samples and 89 normal samples, were retrieved from TCGA (https://xenabrowser.net/). A total of 221 RNA expression profiles of HCC from the GSE14520 cohort and 95 profiles from the GSE76427 cohort were obtained from the GEO database (29, 30). The International Cancer Genome Consortium—Liver Cancer–RIKEN Japan (ICGC-LIRI) and related phenotype information were downloaded from HCCDB v2.0 (http://lifeome.net:809/#/home) (31). In addition, the paraffin-embedded liver cancer tissue samples during general surgery were collected in the Mengchao Hepatobiliary Hospital of Fujian Medical University from January 2024 to December 2024. The study was conducted in accordance with the Declaration of Helsinki, and the study protocol was approved by the Institutional Review Board of the Mengchao Hepatobiliary Hospital of Fujian Medical University on November 3, 2023.

### 2.2 Single-cell RNA sequencing

The Seurat (version 4.4.0) package was used for processing and further analysis of data (32). Single-cell gene expression profiles were filtered using the criteria of a minimum threshold of 200 genes and a maximum threshold of 20% mitochondria genes per cell. The top 2,000 high variant genes were identified and scaled with the ScaleData function. To eliminate batch effects, all cells from the PT, PVTT, and MLN were integrated using Harmony (33, 34). The parameter setting in the Harmony algorithm was as follows: group.by.vars = “orig.ident”, max.iter.harmony = 10, lamba = 1. Using the RunPCA, FindNeighbor, and FindClusters functions, principal component analysis (PCA) linear dimensionality reduction and cluster visualization were performed. Cell clusters were annotated based on the cell lineage-specific genes (20, 28, 35), such as *CD3D*, *CD3E*, and *CD3G* for T/natural killer (NK) cells; *PECAM1*, *DCN*, and *TM4SF1* for endothelial cells; *ACTA2*, *DCN*, and *COL1A2* for fibroblasts; *MS4A1*, *CD79A*, and *MZB1* for B/plasma cells; *LYZ*, *CD14*, and *CD68* for myeloid cells; and *ALB*, *SERPINA1*, and *KRT8* for hepatocytes. Sub-cluster analysis of myeloid cells was conducted with the SingleR package. According to previous documents, two different TAM clusters were identified based on specific cell type genes, including SPP1+ TAMs (*SPP1*, *TREM2*, *FABP5*, and *NUPR1*) and FOLR2+ TAMs (*CD163*, *FOLR2*, *C1QB*, and *SEPP1*) (13). These annotations were confirmed using the random forest algorithm. The “FindMarkers” function in Seurat was used to determine the differentially expressed genes (DEGs) between the two clusters. The non-parametric Wilcoxon rank-sum test was used to obtain *p*-values for comparison. The Monocle2 package was utilized to conduct pseudotime trajectory analysis of the TAMs for cell-to-cell variant demonstration (36). Genes along the trajectory were obtained and were enrolled in subsequent patient classification.

### 2.3 Identification of TAM-related subtypes

Non-negative matrix factorization (NMF) is an algorithm that can reduce high-dimensional datasets of tens of thousands of genes into a handful of metagenes, which are biologically easier to interpret (37). NMF has been widely used in various fields such as image analysis, speech recognition, auto signal processing, and bioinformatics (38). Using univariate Cox regression, prognosis-related genes were filtered from the trajectory genes. Subsequently, the consensus matrix of the filtered genes and clustering were constructed with the NMF algorithm. The parameter settings in the NMF algorithm were as follows: factorization ranks = 2–10, methods = “lee,” number of runs = 100 (38). The optimal rank was determined according to the cophenetic coefficient, before which a sharp decrease was observed (38). The silhouette graphic was used to qualify the clustering robustness. Specifically, individual silhouette statistics ranges from −1 to +1, where a high value indicates that the object is well matched to its own cluster and poorly matched to neighboring clusters (39).

### 2.4 Exploration of cluster-specific biological characteristics

Analysis of the DEGs between groups was performed using the DESeq2 package. The criteria log2 |foldchange| > 2 and *p* < 0.01 were used to filter genes. With the sorted expression profile, gene set enrichment analysis (GSEA) was conducted to elucidate the biological characteristics of distinct groups. The C2.cp.kegg.v5.2.symbols.gmt from the Kyoto Encyclopedia of Genes and Genomes (KEGG) pathway database was selected as the reference gene set. Hub gene-associated functional pathway enrichment was performed using the Gene Ontology (GO) pathways.

To evaluate the relative abundance of SPP1+ and FOLR2+ macrophages within the tumor and adjacent normal tissues, the DEGs [filtered using log2 (foldchange) > 0.585 and *p* < 0.01] in these two cell types were used as signature gene sets. Thereafter, gene set variation analysis (GSVA) (40) was applied to calculate the GSVA scores of the SPP1+ and FOLR2+ macrophages in TCGA and other independent datasets. Moreover, to explore the specific functional pathways of distinct macrophage clusters, the GSEA algorithm was performed using the R package clusterProfiler. The DEGs were determined using the “FindMarkers” function in the R package Seurat as described above.

### 2.5 Weighted gene co-expression network analysis

Weighted gene co-expression network analysis (WGCNA) is commonly used to reveal the patterns of gene expression, summarizing the interconnections between modules and clinical traits, as well as identifying the candidate biomarkers or therapeutic targets (41, 42). In this study, cluster-associated regulated genes were investigated using the WGCNA package. Specifically, genes with the top 25% variance were selected and outlier samples were removed. By choosing an optimal soft threshold *β* (*β* = 5), the correlation matrix was converted into an adjacent matrix, and a topological overlap matrix (TOM) was subsequently formed. Utilizing average linkage hierarchical clustering, the 1 − TOM dissimilarity metric categorized genes with similar expression into gene modules. With the dynamic tree cut function, gene modules with diverse colors were determined. Finally, based on the relationship between modules and clinical traits, cluster-related modules with the tightest correlation were identified and signature genes were accordingly extracted.

### 2.6 Hub gene screening and visualization

The STRING website (https://cn.string-db.org/) is widely used for exploring protein interaction networks. Overlapping genes between the signature genes and the DEGs [filtered using log2 |foldchange| > 2 and *p* < 0.01] were submitted to the STRING website, and then the relative interaction network was retrieved, which was further imported into the Cytoscape software for visualization. The maximal clique centrality (MCC) algorithm in the cytohubba plug-in was used to obtain the top 10 ranked nodes as hub genes. Another plug-in, molecular complex detection (MCODE), was used to screen the hub modules (degree = 2, node score cutoff = 0.2, *k*-core = 2, max.depth = 100). A Venn diagram was used to capture an overlap of the hub nodes that were determined by the two algorithms mentioned above.

### 2.7 Immune cell infiltration analysis

xCell is a gene signature-based tool that utilizes a large compendium of publicly available transcriptomic data to infer the enrichment scores of 64 immune and stromal cell types (43). Using the xCell algorithm, the abundance of the adaptive immunity cells within each sample was quantified. Comparative analyses were performed to assess differential immune cell infiltration patterns across distinct clusters. Correlation analyses were conducted to investigate the relationship between the abundance of the adaptive immune cells and the expression levels of the cluster-specific signature genes.

### 2.8 Multiplexed immunofluorescence staining

Tumor tissues were first fixed in 10% formalin, embedded in paraffin, and then serially sectioned to 4-µm thickness. The following primary antibodies (all from ServiceBio, Wuhan, China) were used: CD8 (dilution 1:2,000), CD68 (dilution 1:5,000), and SPP1 (dilution 1:5,000). Subsequently, the samples were incubated with primary antibodies, followed by secondary antibodies. Double staining of CD8 with SPP1 and CD68 with SPP1 was mutually conducted. 4′,6-Diamidino-2-phenylindole (DAPI) was used for visualization of the cell nuclei (ServiceBio, Wuhan, China). A Nikon ECLIPSE C1 microscope was used for all imaging.

### 2.9 Somatic mutation analysis between clusters

The maftools package was utilized to identify and depict the top 10 somatic mutations among different clusters, including single nucleotide polymorphisms (SNPs), insertions and deletions (INDELs), the tumor mutation burden (TMB), and the mutation frequency (44).

### 2.10 Drug response prediction and potential therapeutic agent identification

Tumor Immune Dysfunction and Exclusion (TIDE) is commonly used to predict the immune-escape probability to comprehensively evaluate the T-cell infiltration and the T-cell function status. The TIDE score is available through the online algorithm (http://tide.dfci.harvard.edu/). In general, higher TIDE scores are associated with a poorer immune checkpoint inhibitor (ICI) therapeutic effect (45). The oncoPredict is an R package used to predict drug response and potential biomarkers based on cell line screening data (46). Importing the training matrices (GDSC and CTRP) from the website (https://osf.io/c6tfx), the sensitivity of the different clusters to chemotherapeutic or targeted drugs was predicted. The connectivity map (CMap) is a powerful tool based on pattern-matching algorithms for the identification of potential therapeutic compounds for specific populations (http://clue.io/) (47). The overlapping genes between the signature genes and the DEGs [filtered using log2 |foldchange| > 2 and *p* < 0.01) were selected and then the similarity of their expression profiles compared using the CMap database. Finally, the top 10 compounds from each cluster were obtained according to the ascending negative scores.

### 2.11 Statistical analysis

The Kruskal–Wallis test was used to compare the DEGs among the three groups. The survival probability between clusters was compared with a log-rank test. The Kaplan–Meier and Cox regression analyses were performed with the survival R package. Independent risk factors were identified using the multivariate Cox proportional hazards regression model. All statistical analyses were performed with R version 4.2.2.

## 3 Results

### 3.1 Single-cell transcriptomic atlas and myeloid cell landscape of HCC

**Figure 1:**
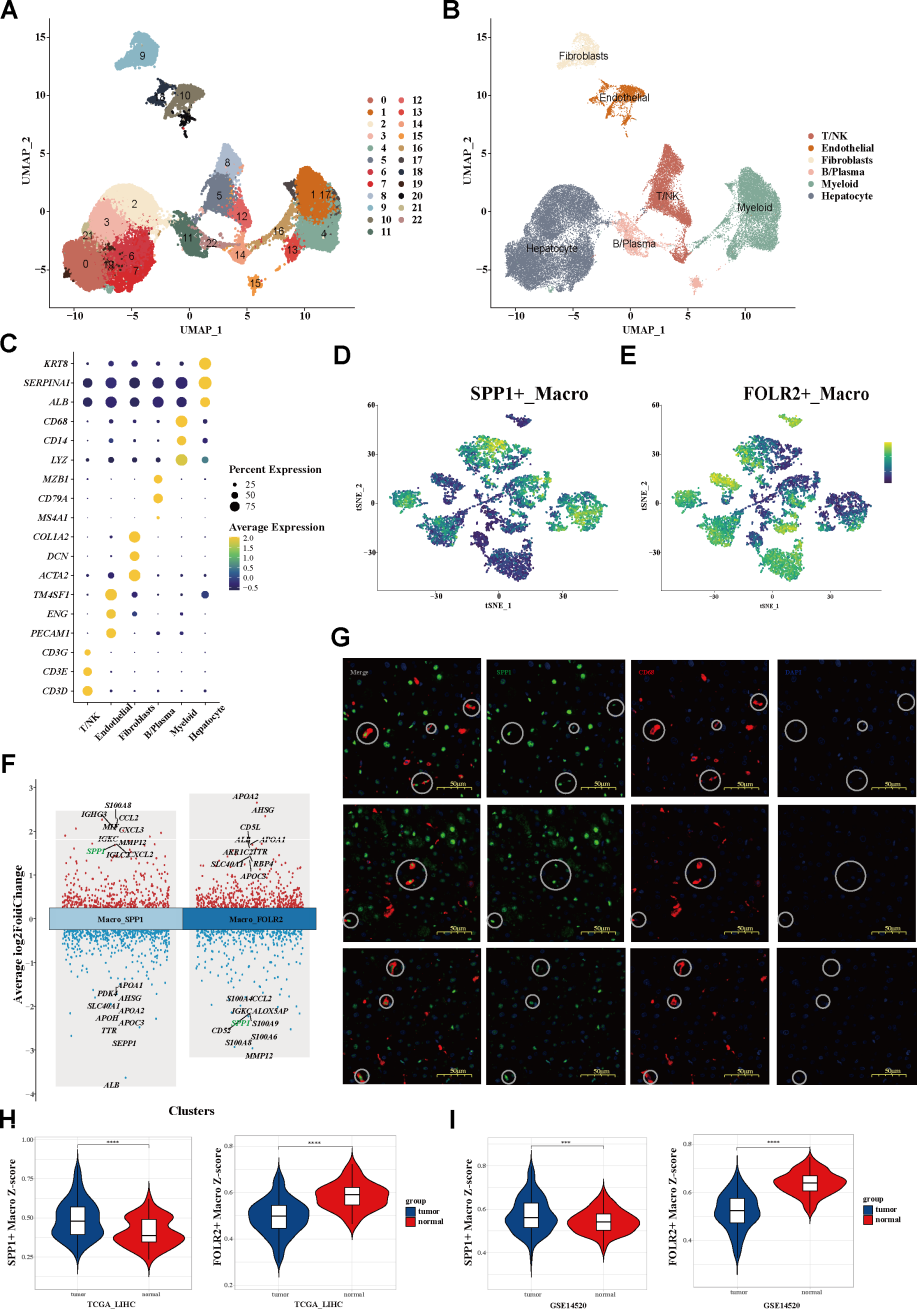
Single-cell transcriptomic atlas of the global microenvironment and myeloid cell landscape of HCC. **(A, B)** UMAP plot of a total of 23 clusters **(A)** and six main cell types **(B)** from three tissue sites of 10 patients with HCC. **(C)** Dot plot showing the percentage of expressed cells and average expression levels of the canonical markers among six major cell types. *Circle sizes* represent the percentage of cells within a cluster expressing a gene. *Color* represents the average expression of each gene. **(D, E)** tSNE plots **(D)** and the mutually exclusive expression of the SPP1 and FOLR2 signatures **(E)** within myeloid cells. **(F)** Top 10 upregulated and downregulated genes of the two clusters. **(G)** mIHC staining determining the distribution of SPP1+ macrophages. **(H, I)** Signature scores of SPP1+ macrophages **(H)** and FOLR2+ macrophages **(I)** between tumor and adjacent normal tissues. *HCC*, hepatocellular carcinoma; *UMAP*, uniform manifold approximation and projection; *tSNE*, t-distributed stochastic neighbor embedding; *mIHC*, multiplex immunohistochemistry.

To depict the landscape of the global microenvironment of HCC, single-cell transcriptomic analysis was performed on tumor sections from 10 patients, including 10 PTs, two PVTTs, and one MLN (**Supplementary Table S1**). After log-normalization and dimensionality reduction, a total of 43,228 cell transcriptomes were classified into 23 clusters (**Figure 1A**). According to the respective canonical markers, six main cell types—hepatocytes, T/NK cells, B/plasma cells, myeloid cells, fibroblasts, and endothelial cells—were defined (**Figures 1B, C**; **Supplementary Figures S1A–D**; **Table S2**). To further investigate the TAMs, a total of 8,038 myeloid cells were filtered out and categorized into three subpopulations: macrophages, monocytes, and dendritic cells (DCs) (**Supplementary Figure S1F**). Significantly, beyond the conventional M1/M2 macrophage dichotomy, two distinct macrophage phenotypes characterized by SPP1+ macrophage and FOLR2+ macrophage signatures were identified at the single-cell level. The t-distributed stochastic neighbor embedding (tSNE) map was used to delineate the segregated macrophage clusters, composed of the SPP1+ TAM and FOLR2+ TAM subsets, which exhibited mutually exclusive and DEG signatures (**Figures 1D, E**; **Supplementary Figures S2F–H**). These subset annotations were confirmed by analysis of the DEGs (**Figure 1F**) and the random forest algorithm (**Supplementary Figure S2I**). *SPP1* emerged as one of the top upregulated genes in SPP1+ TAMs, but was significantly downregulated in FOLR2+ TAMs. Tumor samples were histologically accessed in order to gain insights into the distribution of SPP1+ macrophages in our cohort. Specifically, both SPP1+ microphages (with co-localization of CD68 and SPP1) and SPP1− macrophages (CD68^+^ only) were observed (**Figure 1G**).

### 3.2 Trajectory analysis revealed TAM variations

**Figure 2:**
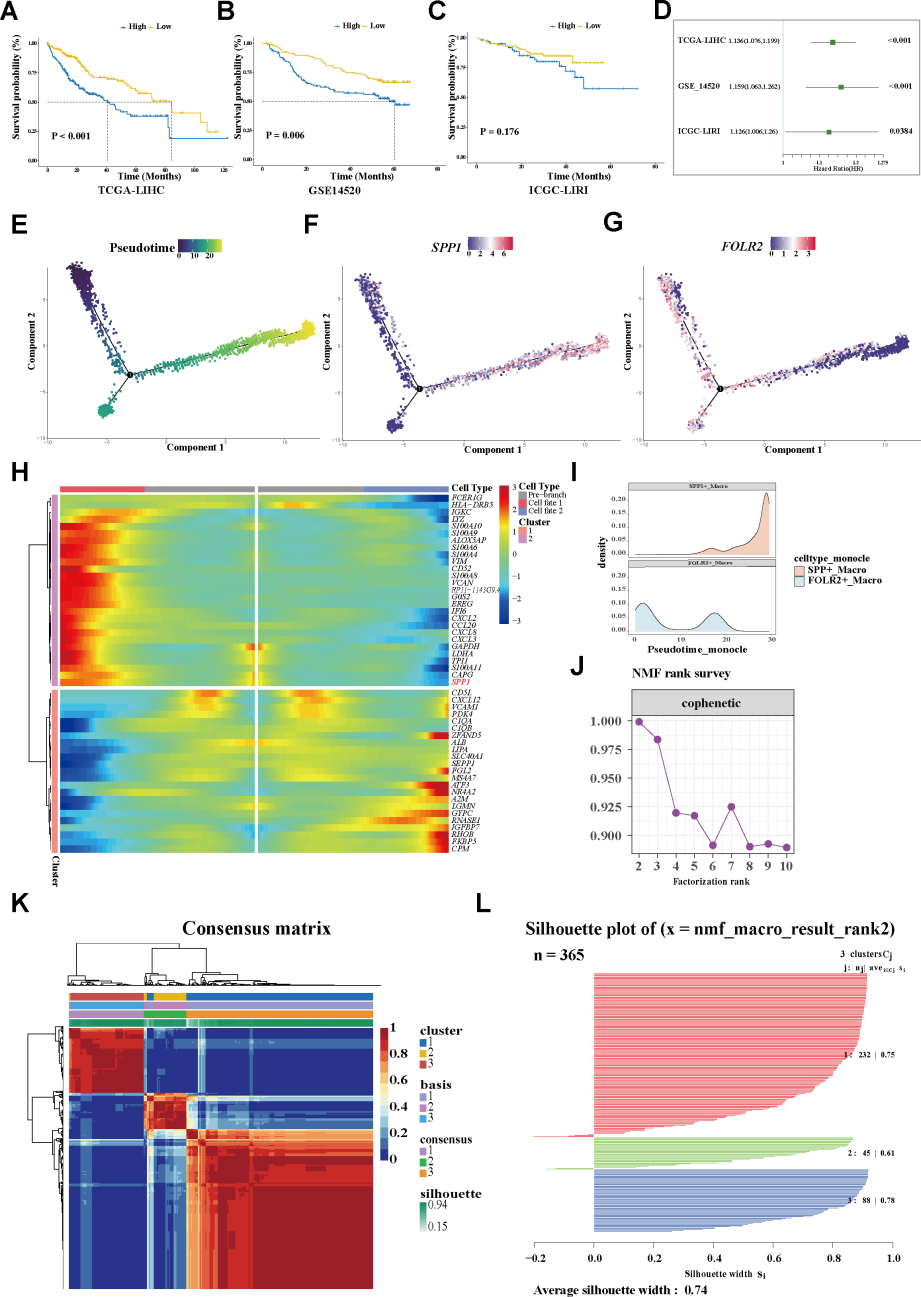
Trajectory analysis revealing variations in TAMs. **(A–C)** Plots of the Kaplan–Meier overall survival curves from TCGA-LIHC **(A)**, GSE14520 **(B)**, and ICGC-LIRI **(C)** cohorts, grouped with high and low expression of SPP1. **(D)** Univariate analyses of SPP1 within the different datasets. **(E)** Pseudotime trajectory showing the distribution of macrophages. **(F)** The SPP1 signature accumulated with the progress of pseudotime. **(G)** FOLR2 signature enriched in the early trajectory stage. **(H)** Heatmap displaying the genes along with a dynamic trend. **(I)** Peak map showing the distribution of the SPP1+ and FOLR2+ TAMs during the pseudotime transition. **(J)** Identification the SPP1+ TAM-related population. **(J)** Cophenetic curve revealing 3 as the optimal rank. **(K)** Consensus matrix map of the NMF clustering of the HCC cases from the TCGA-LIHC cohort. **(L)** Silhouette plot validating the robustness of the NMF cluster. *TAMs*, tumor-associated macrophages; *NMF*, non-negative matrix factorization; *HCC*, hepatocellular carcinoma; *TCGA-LIHC*, The Cancer Genome Atlas—Liver Hepatocellular Carcinoma; *ICGC-LIRI*, International Cancer Genome Consortium—Liver Cancer–RIKEN Japan.

Using the GSVA algorithm, SPP1+ macrophages were found to be significantly increased in tumor tissues, while FOLR2+ macrophages were enriched in adjacent normal tissues (**Figures 1H, I**; **Supplementary Figures S2A, B**). It was further observed that higher expression levels of *SPP1* were remarkably associated with shorter overall survival (OS) in independent HCC cohorts (**Figures 2A–D**). Distinct functions of the two subtypes were also revealed. SPP1+ macrophages highly expressed metalloproteinases (e.g., *MMP12*), macrophage migration inhibitory factor (e.g., *MIF*), and chemokines (e.g., *CXCL2* and *CXCL3*). On the other hand, FOLR2+ macrophages showed higher expression of *CD5L* and the metabolism-associated genes (e.g., *APOA2*, *APOA3*, and *AHSG*) (**Figure 1F**). Functional analysis showed that the chemokine signaling pathway, cytokine–cytokine receptor interaction, glycolysis/gluconeogenesis, the IL-17 signaling pathway, and the PPAR signaling pathway were enriched in SPP1+ macrophages, while complement and coagulation cascades, cholesterol metabolism, and metabolism of xenobiotics by cytochrome P450 were highly enriched in FOLR2+ macrophages (**Supplementary Figure S2C**).

For in-depth exploration of the phenotypic dynamics of the macrophages and their correlated clinical relevance, the Monocle algorithm was applied, considering cells, not clusters, as a unit of statistical replication (27). Notably, it was found that the *SPP1* expression progressively accumulated along a pseudotime trajectory, while the *FOLR2* signature was predominantly enriched in the early stage (**Figures 2E–I**). Within the trajectory, molecules with similar dynamic trends were gathered and displayed in a pseudotime heatmap. The genes along the trajectory were extracted and two coherently regulated gene modules were identified (**Figure 2H**). Notably, *SPP1* was determined as one of the main dynamic genes (**Figure 2H**; **Supplementary Table S3**; the heatmap shows the top 50 trajectory genes).

### 3.3 Identification of the SPP1+ TAM-related population

**Figure 3:**
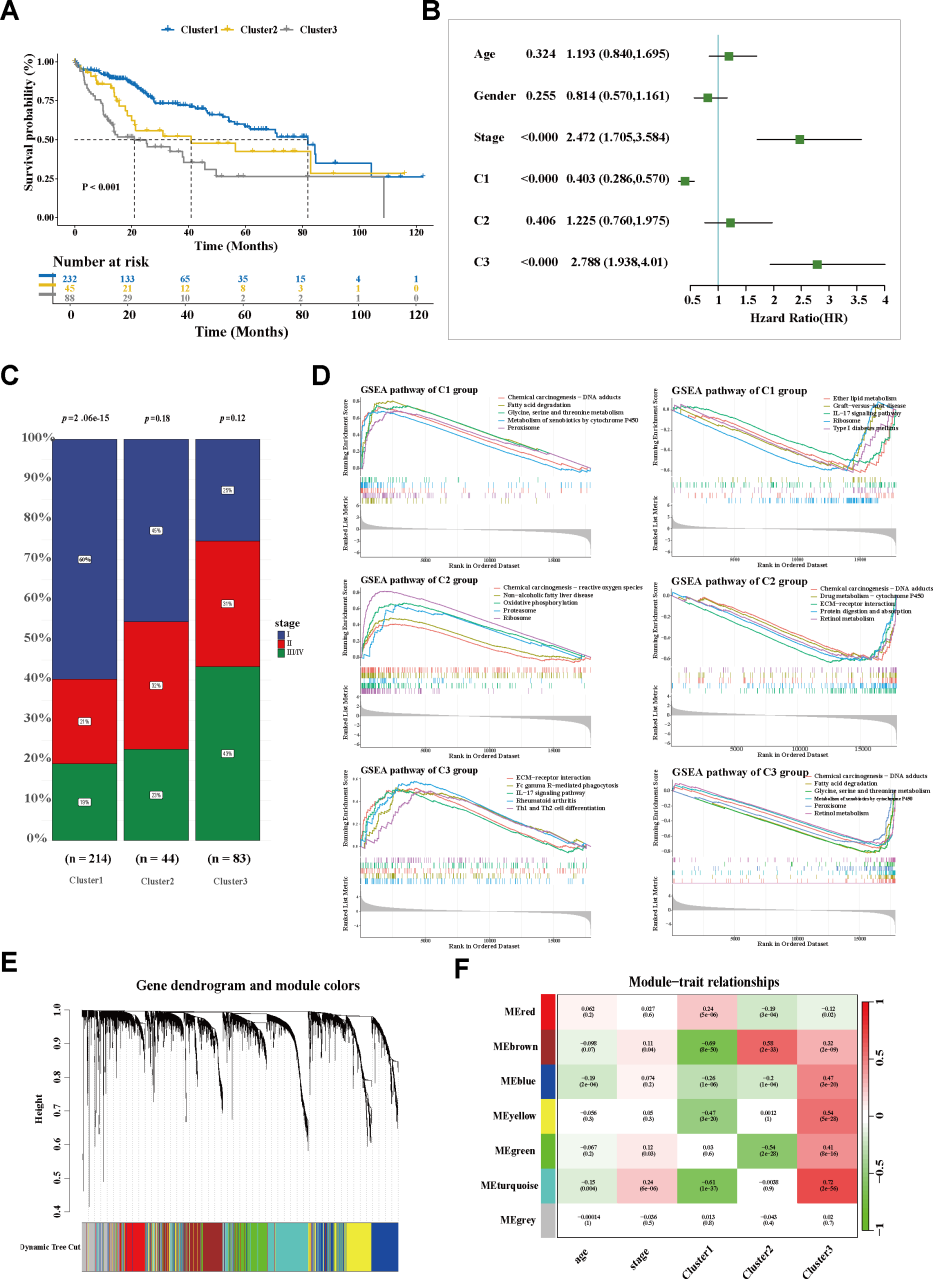
Exploration of the SPP1+ TAM-related population. **(A)** Kaplan–Meier curves of the overall survival of three subgroups in TCGA-LIHC. **(B)** Forest plot of the survival-related factors. **(C)** Bar plot displaying the correlation between HCC stage and cluster stratification. **(D)** GSEA showing the enriched signaling pathways of each cluster. **(E)** The Dendrogram displaying the gene modules with diverse colors. **(F)** Correlation analysis of module eigengenes and molecular phenotypes. *TCGA-LIHC*, The Cancer Genome Atlas—Liver Hepatocellular Carcinoma; *HCC*, hepatocellular carcinoma; *GSEA*, gene set enrichment analysis.

For more in-depth insights into the clinical phenotype changes along with dynamic shifts of the macrophage subtypes, a non-negative matrix was constructed according to the prognosis-associated genes filtered from the trajectory genes (**Supplementary Table S4**). For the sharpest decrease in coefficient observed before 4 in the cophenetic arrangement, 3 was determined as the optimal rank (**Figure 2J**). Unsupervised clustering analysis of a total of 365 tumor patients within TCGA-LIHC determined three groups: 232 cases in cluster 1 (C1), 45 cases in cluster 2 (C2), and 88 cases in cluster 3 (C3). Significant heterogeneity of the transcriptome was observed between clusters (**Figure 2K**). The silhouette graphic further provided proof the robustness of the stratification (**Figure 2L**). In addition, survival analysis displayed that C1 yielded favorable OS, while C3 tended to have the poorest prognosis. Using a multivariate Cox regression model, a C3 classification was determined to be an independent risk factor for shorter survival, whereas C1 was deemed as a protective factor for better outcomes (**Figures 3A, B**). Similarly, the bar graph illustrated that C1 and C2 predominantly comprised patients in the early stage (I/II), while the C3 stratification was significantly associated with patients in the advanced stage (III/IV) (*p* < 0.05; **Figure 3C**), suggesting potential tumor progression from the C1 to the C3 phenotype. Moreover, the underlying biological characteristics were delineated between clusters using GSEA. Consistent with the FOLR2+ macrophage signature, C1 exhibited activation of the metabolic pathways including fatty acid degradation (FAD), amino acid metabolism (glycine, serine, and threonine metabolism), and metabolism of xenobiotics by cytochrome P450 (**Figure 3D**). Conversely, these pathways were significantly suppressed in C3. Notably, previous research revealed FAD to be inhibited in multiple cancers and correlated with increased fatty contents in tumors, which impaired the metabolism of fatty acids (FAs) and promoted the pro-tumoral phenotype polarization of TAMs. Although the amino acid pathway is typically aberrantly activated during oncogenesis, its opposite dysregulation may suppress T-cell proliferation and antitumor immunity. For C3, several significantly upregulated pathways, including ECM–receptor interaction and the IL-17 signaling pathway, were noticeably enriched, similarly to the functional profile of SPP1+ macrophages (**Supplementary Figure S2C**). Overall, C1 was characterized as a metabolism-enhanced, FOLR2+ macrophage-related HCC subtype, while C3 was characterized as a metabolism-dysregulated, SPP1+ macrophage-associated HCC subtype. Metabolic alterations and TME remodeling underlie the observed clinical heterogeneity.

### 3.4 Investigation of the hub molecule network embedded in the SPP1+ TAM-related cluster

**Figure 4:**
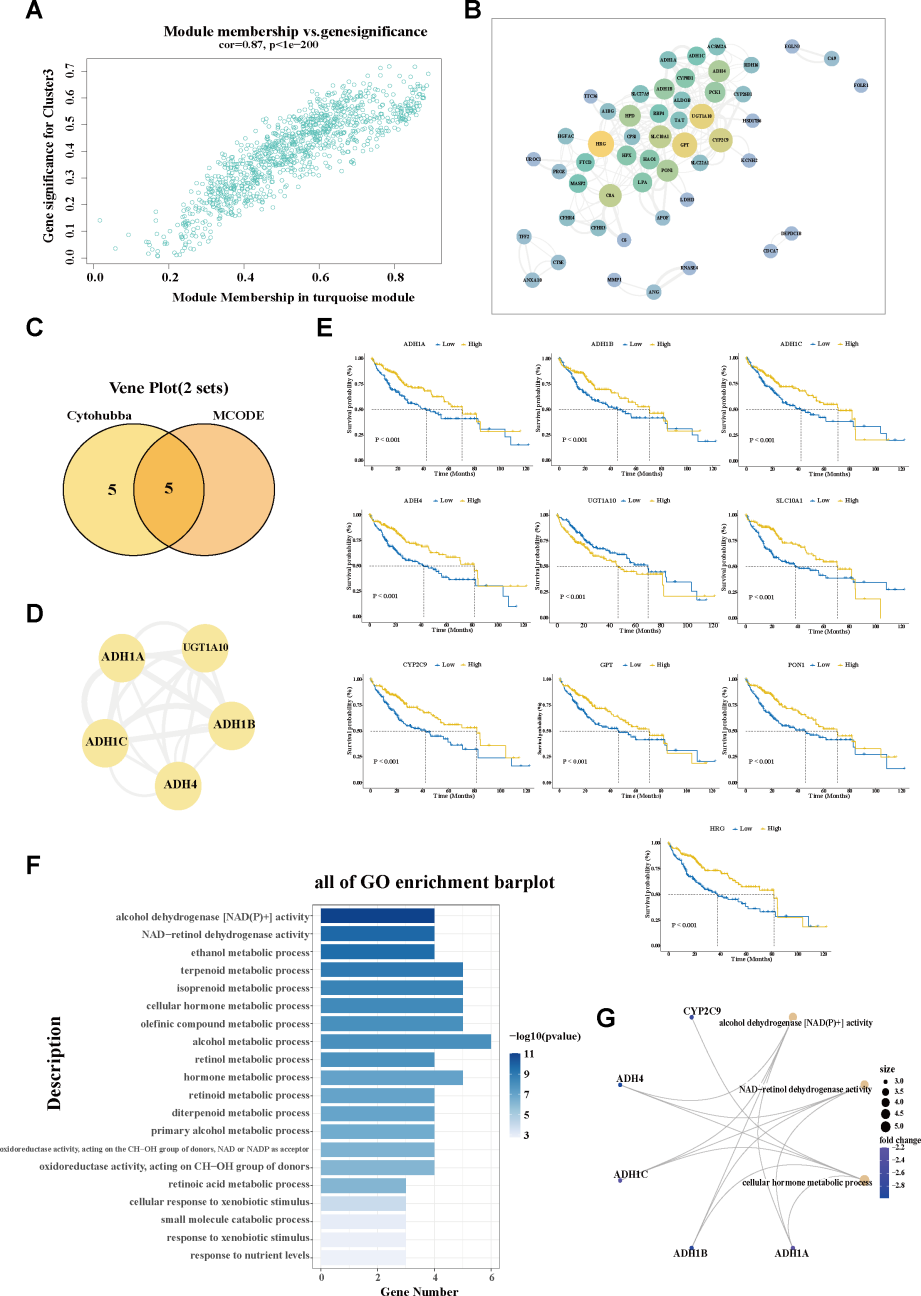
Investigation of the hub molecular network of the SPP1+ TAM-related cluster. **(A)** Scatter plot showing the module membership and gene significance within the turquoise (C3-related) module. **(B)** Interaction network analysis of the genes in the turquoise module using Cytoscape software. **(C)** Venn diagram displaying the overlap of the hub genes and hub module nodes. **(D)** Interaction network of the five overlapping hub genes. **(E)** Correlation of the hub gene expression with overall survival (OS) in The Cancer Genome Atlas—Liver Hepatocellular Carcinoma (TCGA-LIHC). The *blue line* designates samples with lowly expressed genes, while the *yellow line* indicates samples with highly expressed genes. **(F)** Bar plot showing the Gene Ontology (GO) enrichment pathways of the hub genes in the C3-related module. **(G)** The most important hub genes and their corresponding GO pathways. *TAM*, tumor-associated macrophage; *MCODE*, molecular complex detection.

To explore the molecular characteristics of the SPP1+ macrophage-associated subtype, WGCNA was performed. After filtering qualified genes, stratifying all samples, defining an optimal soft threshold *β* (*β* = 5), and transforming the matrix, a total of seven co-expression modules were identified (**Figure 3E**; **Supplementary Figures S3A, B**). The correlations between the gene modules and the clinical traits were determined using a module–trait relationship heatmap (**Figure 3F**; **Supplementary Figure S3C**). The turquoise module exhibited the strongest association with C3. The correlation coefficient between module membership and gene significance validated the robustness of the characteristic genes (**Figure 4A**). Furthermore, the hub genes within the turquoise module were used to construct a gene interaction network using the STRING website (**Figure 4B**; **Supplementary Table S5**). Using the Maximal Clique Centrality (MCC) and Molecular Complex Detection (MCODE) algorithms, hub genes and the core module of the interaction network were identified. Remarkably, a significant overlap was observed between the nodes of the core module and the top 10 hub genes (**Figures 4C, D**; **Supplementary Table S6**), indicating their critical role in this network.

Furthermore, the expression patterns of these hub genes were determined in tumor *versus* paraneoplastic tissues, as well as across C3 and the other subgroups. Specifically, *UGT1A10* exhibited a markedly elevated expression in tumor tissues, while the remaining nine hub genes (*ADH1A*, *ADH1B*, *ADH1C*, *ADH4*, *SLC10A1*, *CYP2C9*, *GPT*, *HRG*, and *PON1*) showed significantly reduced expression levels in cancerous tissues compared with their adjacent normal counterparts (**Supplementary Figure S3E**). A similar trend in expression was also observed when comparing the C3 subgroup with the other subgroups, hinting at the differential expression patterns of these genes in tumor context (**Supplementary Figure S4A**). Based on the expression patterns in cancerous and non-cancerous tissues, the survival analysis revealed that the decreased expression of nine hub genes—*ADH1A*, *ADH1B*, *ADH1C*, *ADH4*, *SLC10A1*, *CYP2C9*, *GPT*, *HRG*, and *PON1*—was significantly associated with worse prognostic outcomes. The elevated expression of *UGT1A10* was correlated with shorter survival time (**Figure 4E**). Data from the Human Protein Atlas revealed that most of the hub genes (i.e., *ADH1A*, *ADH1B*, *ADH1C*, *ADH4*, *CYP2C9*, *GPT*, *HRG*, and *PON1*) belong to the liver-specific enzyme family that is involved in metabolizing various xenobiotic compounds, including alcohol, retinol aliphatic alcohols, hydroxysteroids, and lipid peroxidation products. Pathway analysis also confirmed the involvement of the hub genes in multiple metabolic alterations (**Figures 4F, G**). The downregulation of these hub genes may underlie the characteristic metabolic dysregulation of C3.

Moreover, for C3, which showed a marked enrichment of immune- and inflammation-associated pathways (**Figure 3D**), the potential role of the hub genes in the regulation of immune response was analyzed. Notably, decreased expression of the hub genes was significantly associated with the decreased infiltration of adaptive immune cells, particularly CD8^+^ naive T cells and activated NK cells (**Supplementary Figure S4B**).

### 3.5 Determination of the cluster-driven signature of HCC population stratification

**Figure 5:**
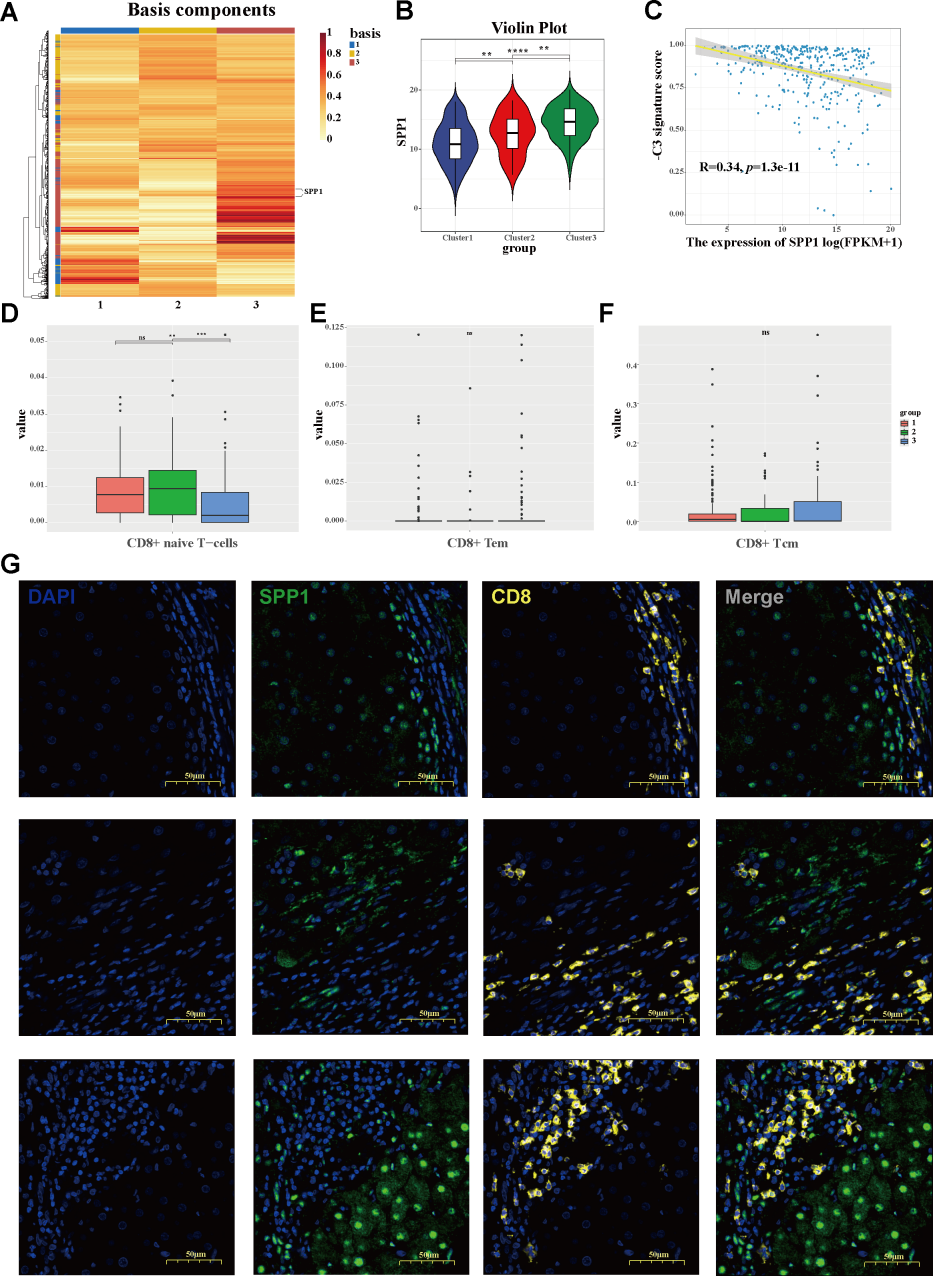
Identification of the cluster-driven signature in C3 hepatocellular carcinoma (HCC) stratification. **(A)** Heatmap depicting the driven signatures of the HCC clusters, with SPP1 emerging as a predominant cluster-driven signature. **(B)** Violin plot of the SPP1 expression levels between clusters. **(C)** Correlation between the expression of SPP1 and the negative cluster score (−C3 signature score). **(D–F)** Bar plots showing the infiltration levels of CD8^+^ naive T cells **(D)**, CD8^+^ effector memory T cells **(E)**, and CD8^+^ central memory T cells **(F)** across three clusters. **(G)** Multiplex immunohistochemistry (mIHC) staining revealing the characteristic localization of SPP1 and CD8 in HCC, with SPP1 tending to suppress the infiltration of CD8^+^ T cells.

To investigate the molecular signatures that drive the HCC population stratification, a heatmap was constructed based on the cluster-related module genes (**Figure 5A**). Exhibiting diverse expression patterns across the HCC groups, *SPP1* emerged as a predominant cluster-driven signature. A remarkably elevated expression of *SPP1* was observed in C3 (**Figure 5B**). Based on the downregulated hub genes within C3, a negative cluster score (−C3 signature score) was calculated to characterize this subgroup. Notably, the expression level of *SPP1* showed a strong correlation with the −C3 signature score (**Figure 5C**), further confirming *SPP1* as a key signature of the C3 subgroup. To investigate the mechanisms underlying the association between the C3 population and poor prognosis, the infiltration of adaptive immune cells was assessed using the xCell algorithm. Intriguingly, the C3 subgroup exhibited a significant reduction in CD8^+^ naive T-cell infiltration compared with the other subgroups (**Figures 5D–F**). Furthermore, utilizing the TIMER 2.0 database, it was demonstrated that the expression of *SPP1* was inversely correlated with the infiltration of both CD8^+^ naive T cells and total CD8^+^ central memory T cells in the HCC environment (**Supplementary Figures S4C, D**). Consistently, multiplex immunohistochemistry (mIHC) staining showed that *SPP1* tended to localize at the tumor boundary, whereas CD8^+^ T cells were likely to localize outside of the tumor, with less infiltration in tumor tissues (**Figure 5G**), hinting at a potential role of *SPP1* in shaping the immunosuppressive microenvironment of HCC and at a promising strategy to counteract immunosuppression.

### 3.6 Identification of subtype-specific therapeutic strategies and promising treatment targets

**Figure 6:**
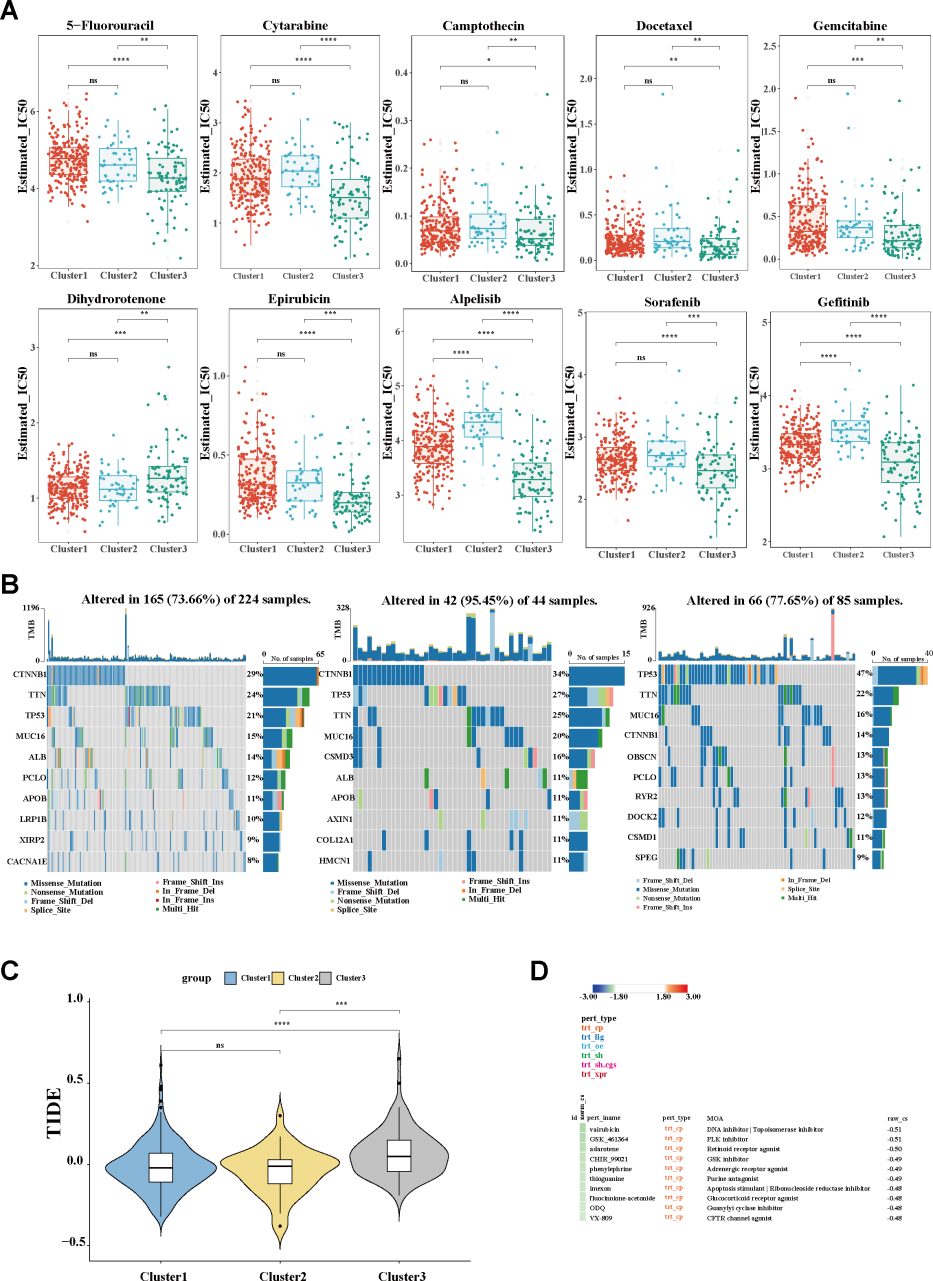
Evaluation of clinical treatment and identification of promising therapeutic targets. **(A)** Box plot of the estimated IC_50_ values of chemotherapy and targeted therapy drugs. **(B)** Waterfall plots showing the distribution of the somatic mutation genes with the top 10 highest mutation frequencies. **(C)** TIDE algorithm for the prediction of the immunotherapy response between clusters. **(D)** Potential therapeutic compounds for the C3 group. *IC_50_*, half-maximal inhibitory concentration; *TIDE*, Tumor Immune Dysfunction and Exclusion.

As distinct molecular phenotypes enable personalized treatment and tailored clinical management for specific HCC subgroups, potential precision strategies for macrophage-associated subtypes were further explored. As is known, transarterial chemoembolization (TACE) remains the preferred treatment for unresectable intermediate-to-advanced HCC. Using the oncoPredict algorithm, the sensitivity of the different clusters for conventional chemotherapeutic drugs was evaluated. As shown in **Figure 6A**, with the relatively lower half-maximal inhibitory concentration (IC_50_), C3 was more sensitive to most chemotherapeutic agents, including 5-fluorouracil, cytarabine, camptothecin, docetaxel, gemcitabine, and epirubicin. The results are in line with the aforesaid stage stratification (**Figure 3C**), wherein C3 was dominated by a population in the advanced tumor stage (III/IV). The sensitivity of the different groups to targeted therapy agents was also determined. Significantly, patients in C3 exhibited superior response to multiple kinase inhibitors, including sorafenib, alpelisib, and gefitinib, compared with those in the other groups. Given the critical role of somatic mutation in shaping cancer phenotypes and the therapeutic response, the mutational landscapes were characterized using the maftools algorithm. Consistent with the established literature, *TP53*, *CTNNB1*, and *MUC16* were identified as the most frequently mutated genes across all subgroups. C3 demonstrated a distinctive mutational profile, with predominant *TP53* mutations (21%, 27%, and 47% positivity, respectively), in contrast to the *CTNNB1* predominance observed in clusters 1 and 2 (29%, 34%, and 14% positivity, respectively) (**Figure 6B**), hinting at the instability of the genome and the dysregulation of the cell cycle in C3. In addition, to further delineate the populations suitable for immunotherapy, the TIDE scores were calculated. A significant increase in the TIDE score was observed in C3, suggesting a poorer response to ICIs. The other two subgroups maintained comparatively lower TIDE scores (**Figure 6C**), implicating a potential group for ICIs. Moreover, using the CMap, potential therapeutic compounds were identified for each cluster (**Figure 6D**; **Supplementary Figures S4A, C**), and correspondingly, the underlying regulatory mechanisms were also elucidated (**Supplementary Figures S4B, D**). Collectively, according to the distinct cluster stratification, personalized treatment strategies would be available and ultimately improve clinical practice.

## 4 Discussion

Although multiple molecular classifications have improved our understanding of the heterogeneity of HCC, the complicated classifier and the reproducible difficulty still pose a substantial challenge in the translation of the subtypes into clinical practice (11, 48, 49). In contrast to previous classifications that focused on comprehensive genomic or transcriptomic analysis, herein, we were concerned on the dynamic evolution of TAMs, potential heterogeneous regulators (14, 15, 50). We specifically identified a novel stratification of TAMs, proposed a simple classification of HCC, and developed personalized therapy correspondingly.

Currently, TMEs with genomic stability and vulnerable nature have increasingly emerged as promising therapeutic targets (12, 13). Multiple associated attempts, including T cells with cytolytic functions or combination therapies that inhibit compensatory signaling pathways, have been developed (9, 51, 52). In this study, using scRNA-seq analysis, we revealed the main cellular components of the TMEs within HCC. We typically focused on the TAMs, pivotal components revealed to govern the cellular and molecular interactions and to sustain hallmarks of cancer, ultimately mapping onto clinical outcomes (14, 15, 50). Similarly to recent single-cell transcription analysis that abandoned the traditional pro-inflammatory M1 and anti-inflammatory M2 dichotomy (50, 53), we failed to clearly distinguish the M1 and M2 macrophages using known markers, such as *CD86* (M1) and *CD163* (M2). Notably, based on previous knowledge (13, 14), we observed a roughly exclusive expression of the *SPP1* and *FOLR2* genes within the macrophage clusters. With regard to the complexity of the cellular ecosystem and the instability of the sub-clusters derived from the dimensionality reduction, we innovatively annotated macrophages into SPP1+ and FOLR2+ TAMs according to simple but distinct variants. We validated the distribution of SPP1+ macrophages in our cohort through immunostaining. Diverse cell states were further defined by trajectory analysis, in which the frequency of *FOLR2* became relatively diluted among TAMs by the expression of *SPP1*. We also observed significant enrichment of the SPP1+ macrophages in tumor tissues compared with adjacent normal tissues, where FOLR2+ macrophages predominated. As FOLR2+ macrophages were revealed to be embryonic-origin tissue-resident macrophages (TRMs, also known as Kupffer cell-like phenotypes in the liver) (50), it was implied that, during tumor progression, SPP1+ macrophages increased from the infiltration of circulating monocytes, accompanied with reduced FOLR2+ TRMs. For functional analysis, higher enrichment of the PPAR pathway was observed in SPP1+ TAMs, and increased expression of the downstream protein metalloproteinases (e.g., MMP12) of this pathway was also identified in SPP1+ macrophages, hinting at a potential function of the activation of the PPAR pathway in the terminal differentiation of SPP1+ TAMs. The dynamic evolution of TAMs may initiate intratumoral cellular program reshaping and heterogenetic clinical phenotype formation. The genes along the trajectory of TAMs provided revealing features that covaried between individuals, which enables a population-oriented molecular classification (54, 55).

Therefore, using the NMF algorithm, heterogeneous molecular clusters of HCC were obtained. Notably, the C3 group was associated with unfavorable prognosis and was regarded as an independent risk factor for worse outcomes. Several upregulated pathways, including the ECM–receptor interaction and the IL-17 signaling pathway, were simultaneously enriched in C3 and SPP1+ macrophages; therefore, C3 was assumed as an SPP1+ macrophage-associated HCC subtype. Significant metabolic alterations, including the downregulated FAD and amino acid metabolism (glycine, serine, and threonine metabolism), were observed in C3. These may be attributed to the reciprocal interactions between the dynamic TAM evolution and the metabolic reprogramming within the TMEs. Similar to *de novo* FA synthesis (56), the inhibition of FAD, as another energy resource, caused FA accumulation in the microenvironment, subsequently inducing dysregulated FA metabolism and promoting pro-tumoral TAM phenotype polarization (57, 58). Furthermore, it is well known that T cells in the TME catabolize lipids through mitochondrial fatty acid oxidation (FAO) to meet the energy demands under nutrient stress (59); however, current evidence demonstrates that unmodified cellular therapy products fail to sustain the bioenergetics in tumors (60). Within increasing FA concentrations due to the inhibition of FAD in the TME, we supposed that a compromised metabolic state of T cells with impaired antitumor effector function is formed (61, 62). Taken together, these mechanisms mobilized the immunosuppressive TME, facilitating tumor progression and proliferation. Although the amino acid pathways are typically upregulated during tumorigenesis, an inverse pattern of dysregulation was observed in the C3 group. This aberrant metabolic reprogramming resulted in altered energy homeostasis, which has been shown to suppress T-cell proliferation and attenuate the antitumor immune response (63, 64).

In addition, most of the hub genes in the C3-associated module belong to liver-specific enzymes and are predominantly enriched in metabolic alteration, which further confirmed the characteristic metabolic dysregulation in C3. Among these hub genes, typically, *ADH1A* is an enzyme that is involved in metabolizing various xenobiotic substrates (65, 66). Evidence suggests that the downregulation of *ADH1A* may facilitate transition from liver damage to hepatocarcinogenesis and exacerbate HCC progression upon exposure to xenobiotic compounds (67). Consistently, a reduction in the expression of *ADH1A* has been observed in hepatocarcinoma tissue compared with para-tumor tissues, and this pattern is also evident in C3 compared with C1 and C2. Similar influences have also been observed in other hub genes within C3, which conclusively contribute to the C3 phenotype formation.

Based on the downregulated hub genes, a negative cluster score (−C3 signature score) was defined for the C3 subgroup of HCC. For the characteristic gene driving the specific molecular classification, *SPP1*, derived from the overlap of the trajectory genes and the main module genes and significantly correlated with the −C3 signature score, was identified as the primary signature of C3. Also known as OPN, *SPP1* is an integrin-binding glycoprotein that has been reported to be overexpressed in various tumors (68). The literature has elucidated the crucial role of *SPP1* in remodeling the TME, including promoting migration and colony formation and facilitating M2-like polarization and immune cell suppression (69, 70). Our findings demonstrate the remarkable increase in the expression of *SPP1* in HCC tissues, which was strongly correlated with a reduced CD8^+^ T-cell infiltration. The mIHC staining revealed that *SPP1* tended to localize at the tumor boundary, whereas CD8^+^ T cells were likely to localize outside of the tumor, further confirming the immunosuppressive role of *SPP1*. Typically, among the three classification phenotypes, the expression of *SPP1* was markedly elevated in the C3 compared with the C1 and C2 subtypes, which may partially explain the immunosuppressive microenvironment within C3. As ICI-based therapy has been established as the first-line treatment and its efficacy is highly dependent on TME (4), targeting *SPP1* may be a promising strategy to regulate the TME and improve immunotherapy response.

As distinct molecular phenotypes uncovered features that covary between individuals, personalized treatment recommendations and tailored management are available for specific groups of HCC. Through the oncoPredict algorithm, the therapeutic efficacy of various treatments across the three subtypes was evaluated. Specifically, C3 exhibited greater sensitivity to most chemotherapeutic agents, including 5-fluorouracil, cytarabine, camptothecin, docetaxel, gemcitabine, and epirubicin. A plausible explanation for this observation may lie in the impaired metabolic pathways within C3, which imposed significant chemotherapeutic stress and influenced the subsequent treatment response (71). Sorafenib is the standard systemic therapy for advanced HCC (4). Notably, the superior efficacy of sorafenib was observed in C3 compared with the other subgroups, along with other kinase inhibitors including alpelisib and gefitinib. Previous studies have elucidated that sorafenib-mediated lipotoxicity contributes to its therapeutic efficacy, in which sorafenib acts as a direct LXR signaling activator and promotes lipogenesis and a toxic accumulation of FAs (11, 52). For patients in C3, the downregulated FAD pathway may have already predisposed them to lipotoxicity stress prior to treatment. The additional administration of sorafenib exacerbated this lipotoxic effect, which ultimately enhanced the therapeutic response in this group. Furthermore, consistent with data indicating that HCC with *TP53* mutation is markedly associated with immunosuppression (67), it was confirmed that the C3 subgroup, which harbored the highest frequency of *TP53* mutations, presented poorer response to immunotherapy. Given the lack of precision medicine in HCC, we identified novel potential therapeutic agents and their corresponding underlying mechanisms based on similar transcription patterns embedded in each cluster. Individualized therapy may be available, accordingly.

Several limitations of the present study need to be acknowledged. Primarily, all of the datasets enrolled in this study were retrospective. Secondly, we provide preliminary evidence that the SPP1+ and FOLR2+ TAMs, rather than the traditional M1/M2 dichotomy, represent a novel macrophage stratification at single-cell resolution. Adequately powered and well-designed studies are required to confirm these findings. In addition, although we revealed the tumor heterogeneity and stratified HCC into three molecular clusters, along with providing potential therapeutic recommendations for specific subgroups, the causality and the precise underlying mechanisms remain to be further elucidated. Population-oriented scRNA-seq analysis is expected for a better understanding and interpretation of our findings.

## 5 Conclusion

We established a novel TAM stratification system and classified HCC populations based on the TAM trajectory genes. *SPP1* was identified as a key signature of the malignant cluster, which negatively impacted immune cell infiltration. Targeting *SPP1* may improve the therapeutic efficacy, and personalized treatment strategies can be tailored based on specific patient stratification.

## Data Availability

Publicly available datasets were analyzed in this study. This data can be found here: The Cancer Genome Atlas portal (https://portal.gdc.cancer.gov/, accession number TCGA-LIHC), and Gene Expression Omnibus (http://www.ncbi.nlm.nih.gov/geo/, accession number GSE149614, GSE14520 and GSE76427), and HCCDB v2.0 (http://lifeome.net:809/#/home, accession number ICGC-LIRI).
