# Supplementary material for: SPP1+ tumor-associated macrophages define a high-risk subgroup and inform personalized therapy in hepatocellular carcinoma
Source: Front Oncol. 2025 Jul 1;15:1606195. doi: 10.3389/fonc.2025.1606195 (PMC12259448; doi:10.3389/fonc.2025.1606195)
Supplement: Supplementary file 7 [file DataSheet1.docx]

Supplementary Material

# Supplementary Figures and Tables

## Supplementary Figures


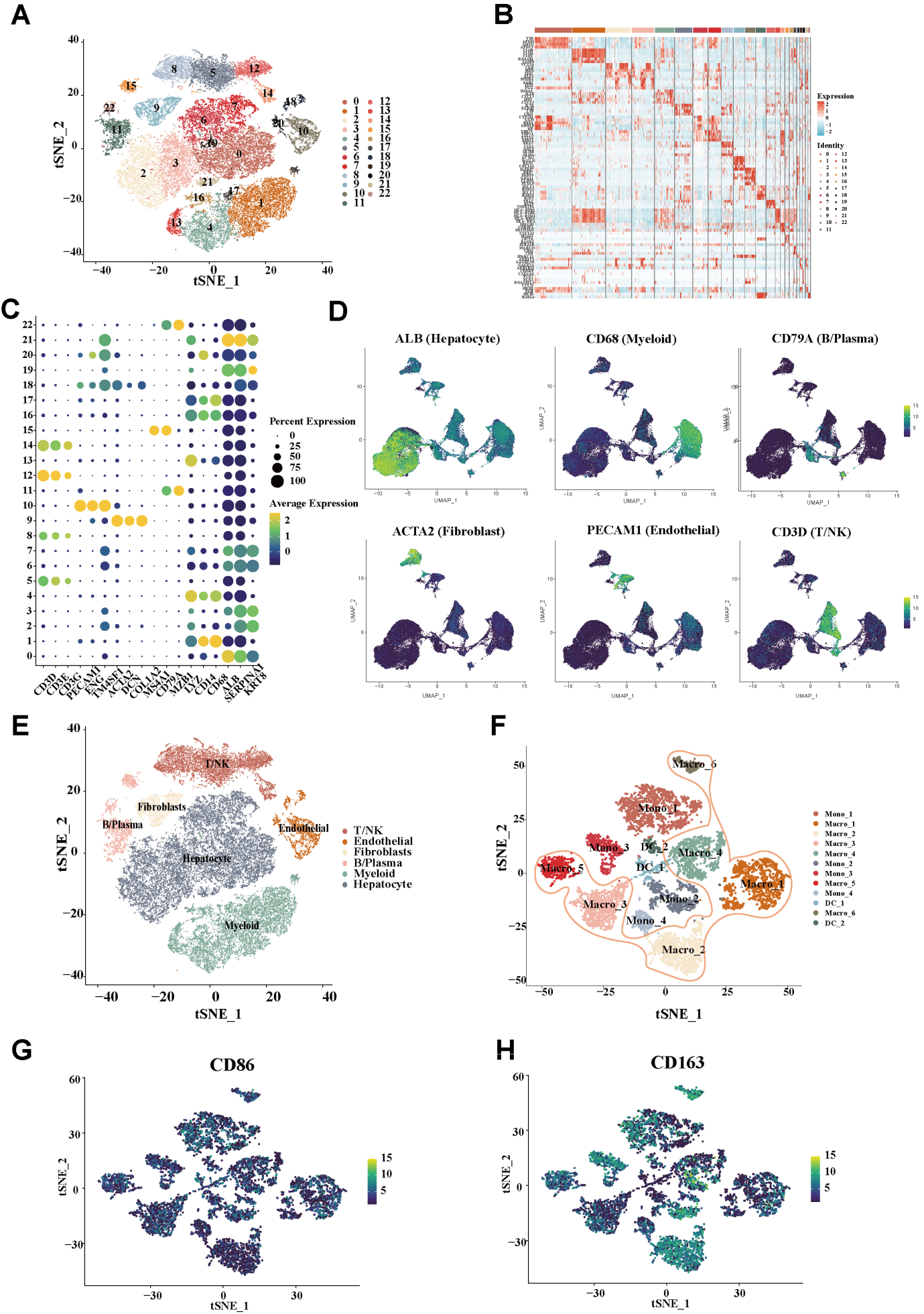


**Supplementary Figure 1.** **Single-cell transcriptomic atlas of global microenvironment and myeloid cell landscape of HCC.** The t-distributed stochastic neighbor embedding (tSNE) of a total of 23 clusters **(A)** and heatmap showing the expression profiles of top 5 most significant genes in 23 clusters **(B)**. Dot plot **(C)** showing the percentage of expressed cells and average expression levels of canonical markers within each cluster. Circles sizes represent percentage of cells within a cluster expressing a gene. Color represents the average expression of each gene. The UMAP plots **(D)** showing the expression levels of specific gene markers of the main cell types. The tSNE plots of the major cell types **(E)**. The t-distributed stochastic neighbor embedding (tSNE) plot **(F)** of 12 clusters of myeloid cells. Macrophages were marked with solid lines. t-SNE plots showing the expression level of characteristic marker genes of M1/M2 macrophages, CD86 **(G)** and CD163 **(H)**.


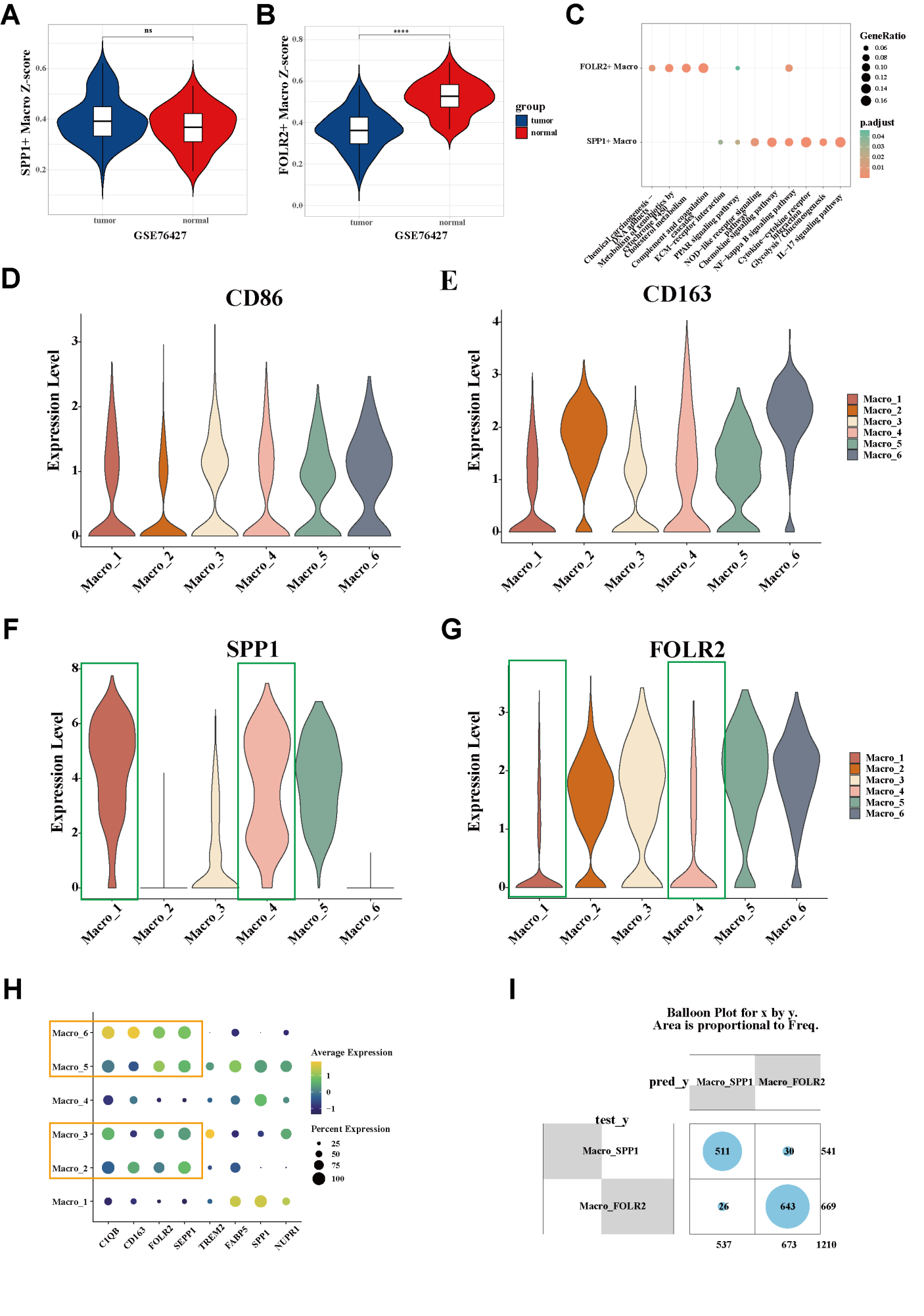


**Supplementary Figure 2 Identification of SPP1+ macrophages and FOLR2+ macrophages.**  Signature scores of SPP1+ macrophages and FOLR2+ macrophages between tumor and adjacent normal tissues **(A)** and **(B)**. Dot plot **(C)** showing the enriched KEGG pathways of differentially expressed genes for macrophage subtypes. Dot size, gene ratio in pathways; dot colour, adjusted p value.Violin plot displaying the expression level of characteristic marker genes of M1/M2 macrophages and SPP1+/FOLR2+ macrophages, including CD86 **(D)** , CD163 **(E)**, SPP1 **(F)** and FOLR2 **(G)**. Dot plot **(H)** showing the expression level of characteristic marker genes across macrophage subclusters. Ballon plot displaying the robustness of the novel classification of macrophages **(I)**.


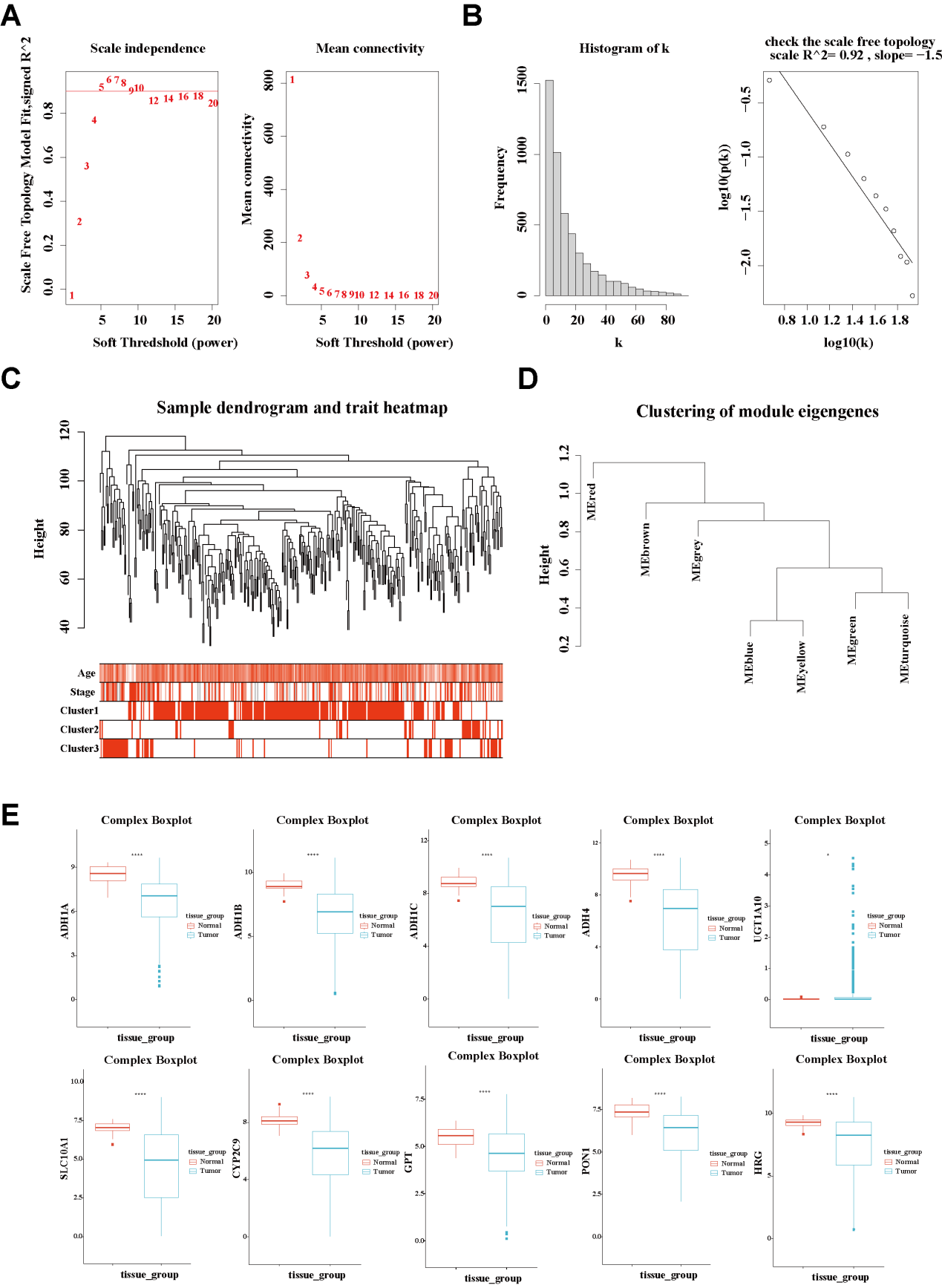


**Supplementary Figure 3 Construction of co-expression modules by weighted genes co-expression network (WGCNA).** Determination of soft-threshold power in WGCNA **(A)**. The left panel showing the impact of soft-threshold power on the scale-free topology fit index; the right panel showing the impact of soft-threshold power on the mean connectivity. Verification of scale free topology when β=5 **(B)**. For right panel, x-axis demonstrating the logarithm of whole network connectivity, and y-axis showing the logarithm of the corresponding frequency distribution. The distribution follows an approximately straight line, termed approximately scale-free topology. Sample clustering dendrogram and trait heatmap **(C)**. Clustering of 7 module eigengenes **(D)**. Box plots showing the expression pattern of hub genes in C3 related module in GSE14520 **(E)**.


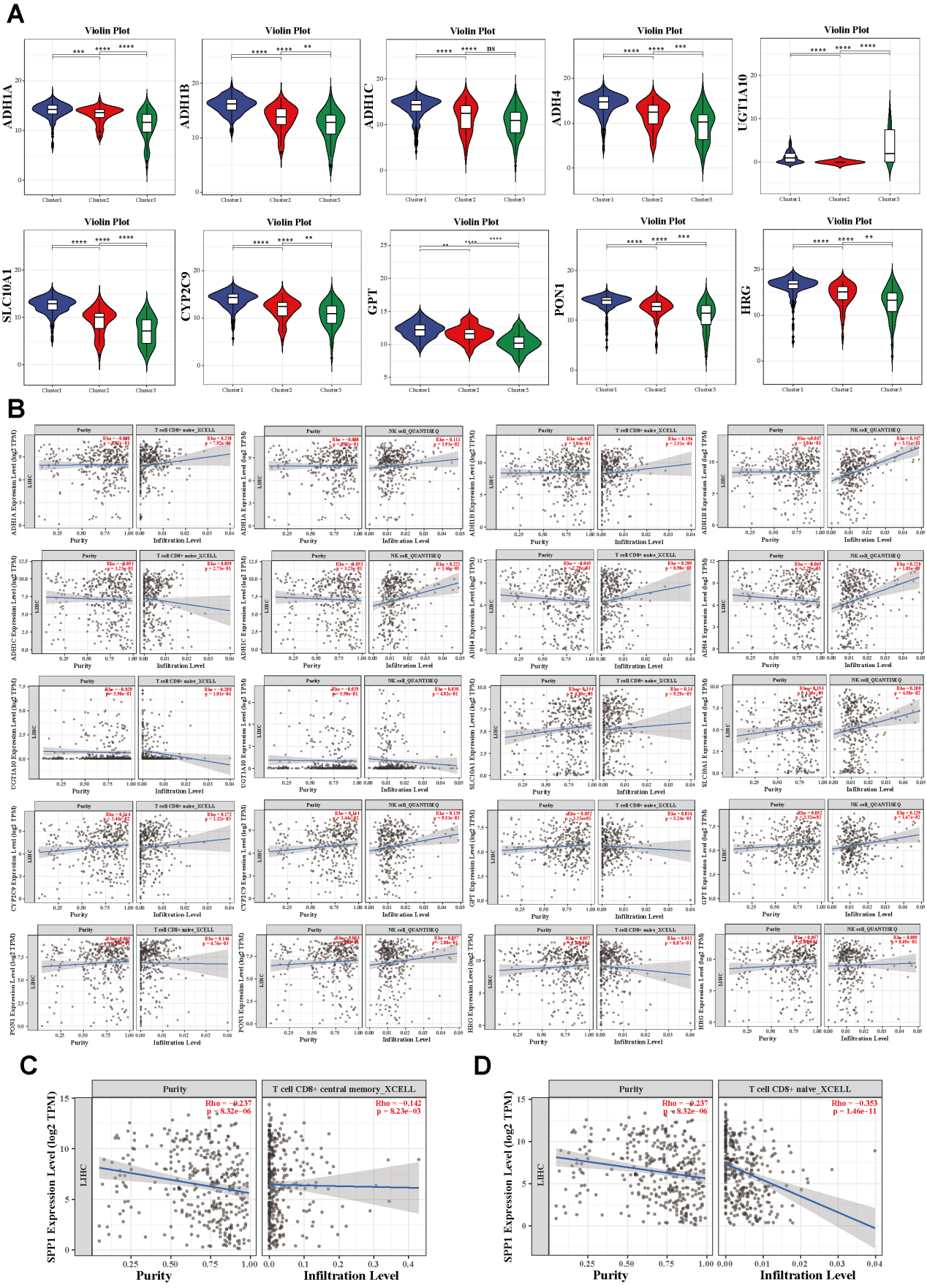


**Supplementary Figure 4 Identification of the expression pattern of hub genes and the relationship between expression level and immune cell infiltration.** Violin plots showing the expression level of hub genes across three subgroups **(A)**. The correlation between the expression of hub genes and the activation of CD8+ naive T cells and NK cells **(B)**. The correlation between the expression of SPP1 and the infiltration CD8+ central memory T cells **(C)** and CD8+ naive T cells **(D)**.


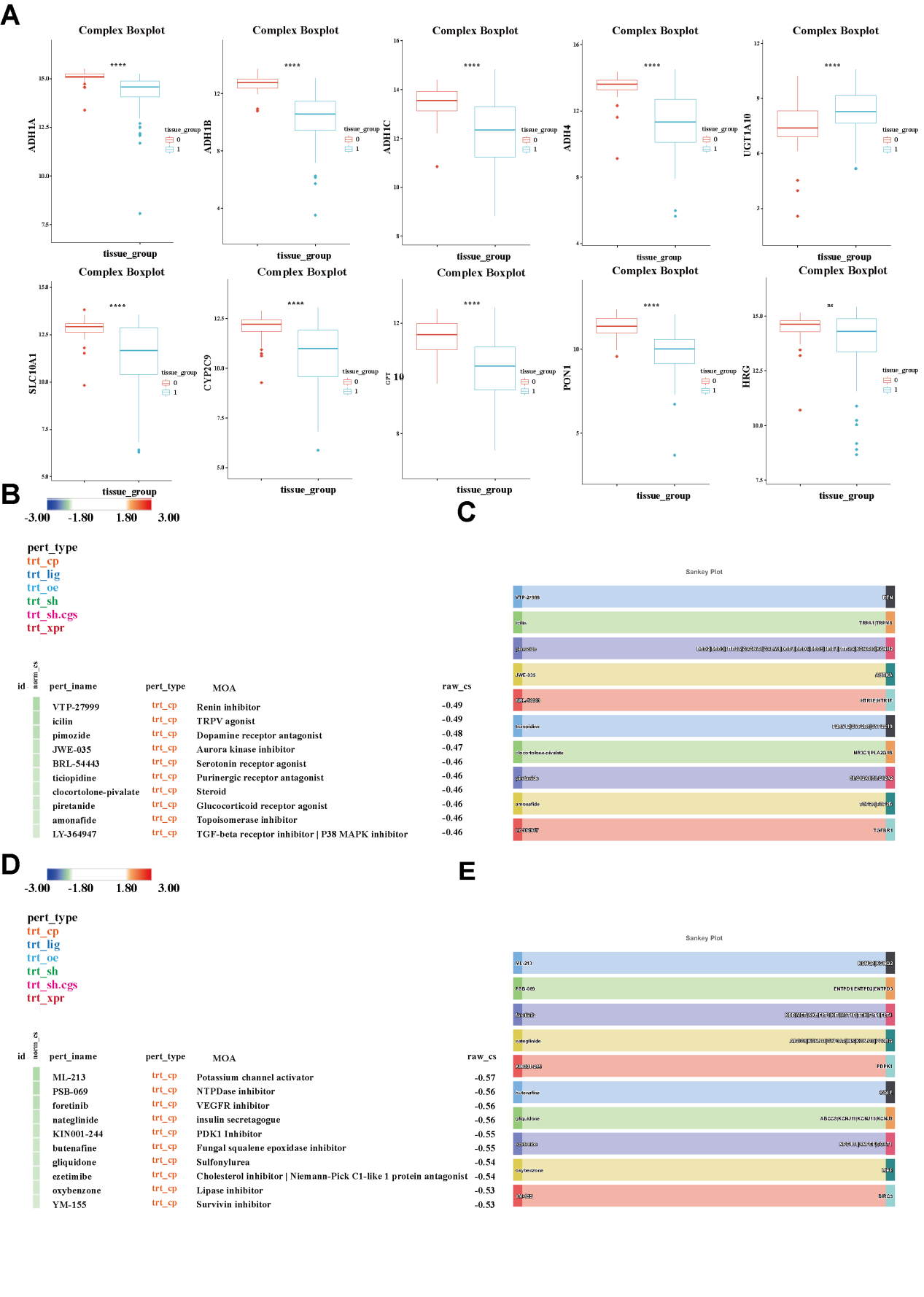


**Supplementary Figure 5 Validation of hub genes expression pattern and exploration of subtype-specific therapeutic targets.** Box plot verifying the expression pattern of hub genes in C3 related module in GSE76427 **(A)**. The potential therapeutic compounds for C1 group and C2 group, **(B)** and **(D)**, and the corresponding underlying mechanisms **(C)** and **(E)**.

**1.2 Supplementary Tables**

**Supplementary Table 1 Clinical characteristics of enrolled HCC patients.**

**Supplementary Table 2 Canonial markers for main cell types.**

**Supplementary Table 3 The trajectory genes enrolled in this study.**

**Supplementary Table 4 Univariate analysises of trajectory genes.**

**Supplementary Table 5 Characteristic genes of cluster-related modules.**

**Supplementary Table 6 Top 10 nod genes and key module genes of C3 interaction network.**

.
